# Distribution of uranium and thorium chains radionuclides in different fractions of phosphogypsum grains

**DOI:** 10.1007/s11356-020-08090-y

**Published:** 2020-02-24

**Authors:** Piotr Szajerski

**Affiliations:** Institute of Applied Radiation Chemistry, Lodz University of Technology, Wroblewskiego 15, 93-590 Lodz, Poland

**Keywords:** Phosphogypsum, Gamma-ray spectrometry, NORM, Radium, Radioactive equilibrium, Isotopes fractionation

## Abstract

This work presents results obtained using gamma spectrometry measurements of phosphogypsum samples on a non-fractionated (native) and fractionated phosphogypsum byproduct. The phosphogypsum was divided into particles size fractions within the range of < 0.063, 0.063–0.090, 0.090–0.125, 0.125–0.250, and over 0.250 mm and analyzed after reaching radioactive equilibrium using high-resolution gamma spectrometry technique. It was found that there is no significant differentiation between ^226^Ra distribution among particular grain size fractions of this material; however, tendency for preferential retention of radionuclides in particular grain size fractions is observed. The detailed analysis of results revealed that radium is preferentially retained in smaller grain size fractions, whereas lead and thorium in coarse fractions. The results indicate that overall ^226^Ra activity concentrations between particular fractions of phosphogypsum vary globally between − 34 and + 47% regarding non-fractionated material, and for ^210^Pb activity concentration, fluctuations are found between − 26 up and + 38%. Presumably, the mechanism of radium incorporation into gypsum phase is based on a sequence of radium bearing sulfate phases formation followed by a surface adsorption of these phases on the calcium sulfate crystals, whereas for lead and thorium ions, rather incorporation into crystal lattice should be expected as more likelihood process.

## Introduction

Phosphogypsym (PG) is a hydrated calcium sulfate, CaSO_4_·xH_2_O, a byproduct generated during wet process of phosphoric acid (H_3_PO_4_) production. It is produced during digestion of phosphate rocks of igneous or sedimentary origin by concentrated sulfuric acid. By now, phosphogypsum is one of the biggest unresolved problem of the chemical industry worldwide. For each metric ton of phosphoric acid, average amount of 4–6 tons of PG is produced (Pérez-López et al. 2007; Tayibi et al. 2009; Rashad 2017; Dvorkin et al. 2018; Machraoui et al. 2019). PG is produced as a dihydrate (CaSO_4_·2H_2_O) or hemihydrate (CaSO_4_·0.5H_2_O), depending on the crystallization temperature (Hodge 1994; El Moussaouiti et al. 1997; EFMA 2000). The dihydrate process can be generalized by Eq. (1).

1 $$ {Ca}_{10}{\left({PO}_4\right)}_6{F}_2+10{H}_2{SO}_4+20{H}_2O\to 10{ Ca SO}_4\cdotp 2{H}_2O+6{H}_3{PO}_4+2 HF\uparrow $$

Globally, phosphoric acid plants have generated about 5 billion metric tons of phosphogypsum, among which 70–90% are deposited in the form of piles (Hilton 2006). This figure is increasing of about 100 to 280 million tons each year (Koopman et al. 1999; Parreira et al. 2003; Reijnders 2007; Biegańska et al. 2013). PG byproduct is often contaminated by acid residues, fluorides, P_2_O_5_, organic impurities (Tranchida et al. 2011), trace and heavy metals (Cd, Pb, Ni, Cu, Mn, Zn, Cr, As and lanthanides) (Koopman et al. 1999; de Oliveira et al. 2007; Pérez-López et al. 2007; Al-Hwaiti et al. 2010a; Tranchida et al. 2011) as well as natural radionuclides. Thus, PG is classified as the NORM residue (Naturally Occurring Radioactive Material), and its utilization in industry is limited to roughly 15% of the total amount produced (Burnett 1995; Rusch et al. 2002; Shen et al. 2007; Pérez-López et al. 2007). Radioactive elements present in PG are mainly ^226^Ra and its progenies (Haridasan et al. 2002). PG is a serious environmental problem and, due to spreading, is able to contaminate locally surface and groundwaters and soil (Rutherford et al. 1994; Poole et al. 1995; Koopman et al. 1999; Haridasan et al. 2002; Othman and Al-Masri 2007; Pérez-López et al. 2007; Al Attar et al. 2011; Jones et al. 2012; Ammar et al. 2013; Wang et al. 2018). The content of radioactive elements in phosphogypsum often exceeds the reference levels regarding ^226^Ra concentration established for building materials (European Commission 2000; Borges et al. 2013; Hassan 2014).

The annual PG production in Poland is estimated for about 1.5 million tons (Biegańska et al. 2013). There are three phosphogypsum stacks in Poland located in different regions: active stack in Police in a north-western Poland operated by Grupa Azoty, Zakłady Chemiczne “Police” S.A. with near 100 million metric tons of PG and two closed stacks in Wiślinka, near Gdańsk (17 million tons) and in Wizów in Lower Silesian region of Poland (3.5 million tons of PG) (Cichy and Jaroszek 2013). Current PG production in Poland is based mainly on the imported phosphorite ores (Cichy and Jaroszek 2013), and PG generated in Polish plants is in 100% stockpiled (Biegańska et al. 2013). So far, many attempts have been made to evaluate and facilitate wider use of phosphogypsum: as building material, soil amendment agent or fertilizer, backfilling and lining materials, and also in chemical industry (Chang 1987; EPA 1992; Rutherford et al. 1994; Guo et al. 2001; Stoulos et al. 2004; Papastefanou et al. 2005, 2006; Değirmenci 2008; Ikhu-Omoregbe 2012; Yang et al. 2012; Hassen et al. 2012; Biegańska et al. 2013; Piątkowska and Pala 2013; Liu et al. 2019). Large-scale application of phosphogypsum as building material is limited mainly because of elevated indoor radiation exposure: both gamma radiation and ^222^Rn inhalation (Papastefanou et al. 2005; Lysandrou et al. 2007; Abril et al. 2009; Bem et al. 2017, 2020; Szajerski and Zimny 2019). However, currently new type of materials are elaborated able to trap efficiently ^222^Rn in a closed pore structures (Gijbels et al. 2018). Utilization of PG as building material is restricted according to the appropriate regulations based on the last implementation of 2013/59/EURATOM Council Directive establishing limits on indoor radiation exposure due to gamma radiation and alpha emitting radionuclides (radon and its progenies) from the naturally occurring radioactive elements (^40^K, ^232^Th and ^226^Ra) in building materials (European Commission 2000, 2014).

The purification of phosphogypsum is difficult, especially in the context of radium isotopes removal. Similar chemistry of radium and calcium makes separation of Ra^2+^ from PG matrix highly complicated and inefficient. So far, the methods proposed for removal of ^226^Ra isotopes from phosphogypsum based on different approaches utilizing extraction, recrystallization, leaching, acid digestion, and combined methods including all above have failed to produce breakthrough and are not implemented in a large scale industrial practice, mainly due to their limited efficiency and high cost intensity (Rajkovic and Vladisavljevic 1997; El-Didamony et al. 2012, 2013a, b; Kovler et al. 2015; Moalla et al. 2017; Moreira et al. 2018; Mashifana 2019).

During production of phosphoric acid, radionuclides are partitioned between H_3_PO_4_ and PG phase. The uranium and thorium isotopes are retained in about 70–85% in extraction phosphoric acid, and ^226^Ra and ^228^Ra, ^210^Pb and ^210^Po coprecipitate with calcium sulfate in phosphogypsum in amounts of about 80–90% (Guimond and Windham 1975; Bolivar et al. 1996; Mazzilli et al. 2000; Bolívar et al. 2009). The most obvious solution for wider PG application in building industry would be its separation into fractions of different radionuclides concentrations, especially concerning ^226^Ra isotope. Such approach would be the most simple solution which does not require expensive chemical processing. So far, there were no complex studies connected with radionuclides distribution between phosphogypsum grains of different sizes. Some results are presented in several papers, where present distribution of ^226^Ra, ^238^U, ^232^Th, ^210^Pb, and ^40^K as well as trace elements between different fractions of phosphogypsum (Arocena et al. 1995; Rutherford et al. 1996; Al-Hwaiti et al. 2010a; Zielinski et al. 2011; Kuzmanović et al. 2020). Generally, there is much more information in scientific literature concerning possible fractionation of non-radioactive trace elements (heavy metals, rare earth elements) than about radionuclides distribution (Arocena et al. 1995; Al-Masri et al. 2004; Al Attar et al. 2011; Rentería-Villalobos et al. 2012).

The main purpose of this study was to verify if there exists any significant difference between ^226^Ra concentration, in fractions of different grain size. Such dependence would make separation of activity more feasible and partially allow for wider utilization of PG after fractionation. The secondary object was an analysis of the existing disequilibrium between main radioactive contaminants in phosphogypsum and their progenies used for quantification of radionuclides in PG.

## Experimental

### Materials

The phosphogypsum samples used in this study were produced in Poland, and were kindly provided by a local phosphoric acid producer. PG was produced from the imported phosphorite ores based on 1/1 (w/w) mixture of Syrian and Tunisian phosphorites (PGA), 1/1 (w/w) mixture of Algerian and Tunisian ore (PGB) and from a pure Tunisian phosphorite (PGC). Phosphogypsym samples were collected directly after their production in phosphoric acid production plant. Unfortunately, there was no possibility for the simultaneous analysis of the phosphorite ore used for phosphoric acid production.

### Samples’ preparation

Phosphogypsum was not modified chemically or physically in any way and used as collected. Prior to sieving, wet phosphogypsum samples were initially dried in the air in the enamel plates during period of about 7 days. After initial drying, samples were further dried in a laboratory drier at 60 °C for 24 h in order to remove water residues. Dried material were kept in tightly closed containers to avoid water capture. After drying, material was subjected to a sieve analysis. Meinzer II Sieve Shaker manufactured by CSC Scientific Company Inc. (Fairfax, USA) has been used for granulometric analysis. Samples of about 650–900 g of phosphogypsum were divided into 6 fractions of different grain sizes: below 0.063, 0.063–0.090, 0.090–0.125, 0.125–0.250, 0.250–0.500, and over 0.500 mm (fractions 0.250–0.500 and > 0.500 were connected), and were named consecutively as F01 – F05 (cf. Table 1). Such sieves’ selection was based on the equipment availability and reasonable considerations about the ease of process implementation in the industrial scale, as further separation of fraction with particles size below 63 μm is much more difficult. From each fraction, about 80 g of phosphogypsum was used for preparation of samples for gamma-ray spectrometry. Samples were prepared as cylindrical plates of 80 mm diameter and about 12 mm thickness by mechanical pressing applying 25 MPa pressure using hydraulic press. After pressing, each sample was placed in a 90 mm diameter polystyrene Petri dish and sealed tightly to prevent radon leaking. The gamma spectrometry measurements were performed at least after 7 weeks (time required for establishing radioactive equilibrium between ^222^Rn and its progenies).

**Table 1:** Phosphogypsum grain size distribution produced from phosphorite blends: PGA – Syrian/Tunisian 1/1 (w/w), PGB – Algerian/Tunisian 1/1 (w/w), PGC – pure Tunisian

Fraction	PG grain diameter, μm	Fractional contribution			Cumulative contribution		
		PGA	PGB	PGC	PGA	PGB	PGC
F01	0–63	0.301	0.246	0.393	0.301	0.246	0.393
F02	63–90	0.292	0.464	0.180	0.593	0.710	0.573
F03	90–125	0.198	0.062	0.069	0.791	0.772	0.642
F04	125–250	0.180	0.193	0.312	0.971	0.965	0.954
F05	> 250	0.029	0.035	0.046	1.000	1.000	1.000

### Radionuclides determination

For the gamma-ray spectrometric measurements, reversed germanium coaxial detector (REGE) was used, type GX2018 from Canberra Industries (Meriden CT, USA). All gamma-ray spectra were collected during 80,000 s inside the 5-cm-thick wall lead shield lined with the 5 mm steel cylinder inside sample chamber. Details of the experimental system are described elsewhere (Bem et al. 2002; Szajerski et al. 2019a, 2019b, 2020). The collected spectra were analyzed using Geenie2000 software. Each time correction for the background radiation was performed taking into account background spectrum collected during 500,000 s. The radionuclides’ activity concentrations were determined according to Eq. (2).

2 $$ {A}_s=\frac{I_s-{I}_{bkg}}{m_s\cdotp {\varepsilon}_{det}\cdotp {\omega}_{em}} $$

where *A*_*s*_ is the activity concentration of particular isotope in a sample, in Bq·kg^−1^, *I*_*s*_ and *I*_*bkg*_ are the *γ*-photons emission rates from sample and from background radiation, in cps, *m*_*s*_ is the net sample weight, in kg, *ε*_*det*_ is the absolute detection efficiency of the measurement system and *ω*_*em*_ is the *γ*-photons emission probability. The absolute (total) detection efficiency for a given geometry was determined using the set of standard reference materials (SRM) from IAEA (phosphogypsum IAEA-434 as well as IAEA-Soil-6, IAEA-327, IAEA-375 and IAEA-368). For the given conditions (cylindrical geometry of 80 mm diameter and 12 mm height), the dependence of *ε*_*det*_ vs. photon energy was determined using the ^226^Ra, ^214^Pb, ^214^Bi, ^228^Ac, ^212^Pb, ^208^Tl, and ^210^Pb photons emission rates from the SRM. The total detection efficiency for a single energy line was determined taking into account the rearranged Eq. (2), which can be described according to Eq. (3):

3 $$ {\varepsilon}_{\mathrm{det}}=\frac{I_{\mathrm{std}}-{I}_{\mathrm{bkg}}}{A_{\mathrm{std}}\cdotp {m}_{\mathrm{std}}\cdotp {\omega}_{\mathrm{em}}} $$

where *I*_std_ is the photon count rate for a reference material and for a single energy line, in cps, *A*_std_ is the certified value of the specific activity concentration for each radionuclide in the SRM, in Bq·kg^−1^, *m*_std_ is the weight of the reference material, in kg, and other symbols are the same as described previously. The measurements and analysis of SRM allowed for determining of the experimental dependence of the total detection efficiency vs. photon energy using an inverse exponential function according to Eq. (4) (Gilmore 2008):

4 $$ {\varepsilon}_{\mathrm{det}}(E)=\frac{1}{B{E}^m+C{E}^n} $$

where *B*, *C*, *m*, and *n* are the empirical fitting parameters used for the global description of the efficiency function *ε*_*det*_ = *f*(*E*, keV) within the investigated photon energy range. No self absorption correction was made for ^210^Pb (46.5 keV) due to the fact that both samples and SRM (IAEA-434) were the same material (calcium sulfate dihydrate of > 95% purity) as well as they were measured in exactly the same geometrical conditions.

The lower limits of detection (LLD) and minimum detectable activity (MDA) were determined according to the well-known Currie’s criterion, and for the given geometry of the detection system, the standard sample weight of 80 g and the typical counting time of 80 ks were determined to be between 0.72 and 7.6 Bq kg^−1^, with the lowest value found for ^228^Th isotope (measured as ^212^Pb, 238.6 keV) and the highest for ^210^Pb (46.5 keV) (Currie 1968). The obtained MDA values were far below the real concentrations of the radionuclides determined during performed measurements.

## Results and discussion

### Phosphogypsum particles size distribution

The posphogypsum samples were analyzed within the grain diameter ranges described in the section “Samples’ preparation”. Sieve analysis of all three samples (PGA, PGB, and PGC) of phosphogypsum revealed that almost all materials fall into particles size range below 250 μm (F01 – F04). These contributions were over 95% with only 2.9, 3.5, and 4.6% fractions of particles size over 250 μm for samples PGA, PGB, and PGC respectively. Because of small contributions of F05 fractions (250–500 and > 500 μm), they were combined into one fraction. Fractions F04 of PGA, PGB, and PGC were slightly inhomogeneous with significant and visible contaminants particulates. Part of these contaminants were of organic origin. Detailed results of sieve analysis together with cumulative contribution calculated are presented in Table 1. All fractions F05 (> 250 μm) contained organic material in amount of ca. 3%. Fractionation of studied phosphogypsum material revealed some differences in particle size distribution for three samples analyzed. Among analyzed samples, for F01 (0–63 μm) fractions, there is nearly 15% contribution variation, from 24.6 (PGB) to 39.3% (PGC). The sample PGA contributes in 30.1% to F01 fraction. Higher dispersion is observed for F02 fractions (63–90 μm range): from 18.0 to 46.4% for PGC and PGB samples respectively. For sample PGB, fraction F02 contribute to 46.4% of the total mass of the sample.

**Fig. 1:**
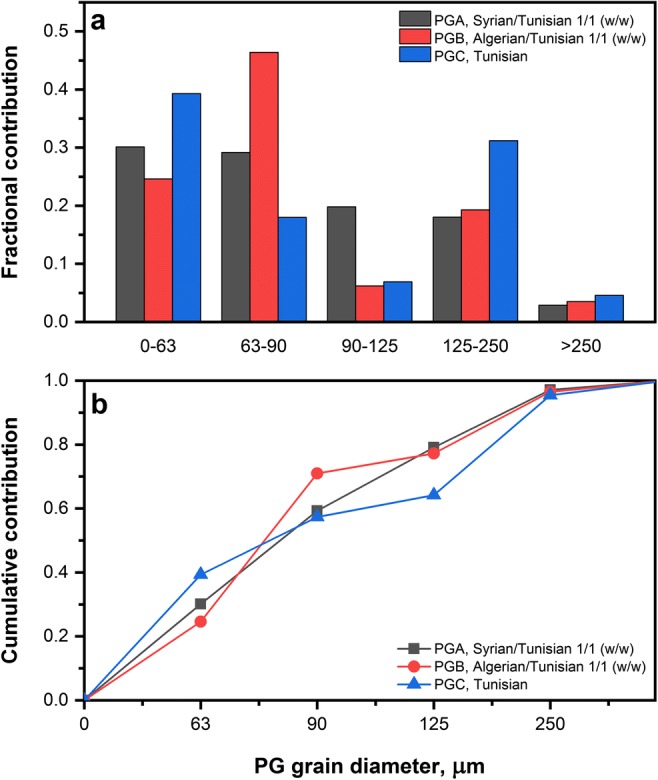
Fractional (**a**) and cumulative (**b**) distribution of phosphogypsum grain size in the PG samples produced from phosphorite blends: PGA – Syrian/Tunisian 1/1 (w/w), PGB – Algerian/Tunisian 1/1 (w/w), PGC – pure Tunisian

The results indicate for a similar contribution of fractions F03 (90–125 μm) for PGB and PGC samples (6.2 and 6.9%) and about three times higher value of 19.8% for the sample PGA. A similar situation is observed in case of F04 fractions (25–250 μm) with 18.0 and 19.3% contribution for PGA and PGB samples and higher (31.2%) contribution concerning sample PGC. There is no significant difference between particular fractions contributions for grains diameter over 250 μm. The graphical representation of the sieve analysis results is shown in Fig. 1.

### Radionuclides concentration in fractionated phosphogypsum

The gamma spectrometry measurements delivered data on the radionuclides concentration in bulk (not fractionated) and fractionated samples of phosphogypsum. Among all radionuclides present in phosphogypsum, the most important are ^238^U series isotopes ^226^Ra and ^210^Pb. The contribution of other isotopes, like ^238^U and ^235^U, is of a minor importance. The input of thorium series radionuclides is of marginal significance, exhibiting only negligible fraction of the total activity related to ^226^Ra and its progenies. It should be expected that during chemical operations with phosphorite ore, isotopes are preferentially separated according to their chemical properties. This results in a breakdown of radioactive equilibrium within uranium and thorium decay chains (Omar et al. 2008; Msila et al. 2016; Jung et al. 2017). Hence, due to the lack of radioactive equilibrium between ^234^Th and ^238^U, it was not possible to use ^234^Th photon emission lines (63.3 and 92.6 keV), and in consequence, activity concentrations of ^238^U must have been estimated taking into account activity of other uranium isotope, ^235^U, assuming constant activity ratio between both uranium isotopes (*A*_*238U*_*/A*_*235U*_ = 21.73). All the results of gamma spectrometry measurements are presented in Table 2 for three PG samples concerning the bulk (not fractionated) and fractionated material. As presented in Table 2, one can see that the activity concentration of ^226^Ra isotope was determined by a two way approach based on both ^226^Ra daughter isotopes ^214^Pb (by its 295.2 and 351.9 keV emission lines) and ^214^Bi (609.3, 1120.3 and 1764.5 keV) and pure 186.2 keV emission from ^226^Ra. Because the 186.2 keV line interferes with 185.7 keV emission from ^235^U, correction applied on 186.2 keV line was based on the relative contribution of both isotopes and ^235^U activity concentration derived from 143.8 keV. The working formula for that can be expressed by Eq. (5),

5 $$ {A}_{226 Ra}^{186}=\frac{I_{186}-{I}_{bkg}}{m_s\cdotp {\varepsilon}_{186}\cdotp {\omega}_{Ra}}-{A}_{235U}\cdotp \frac{\omega_U}{\omega_{Ra}} $$

where *I*_*186*_ and *I*_*bkg*_ are the sample and background photon emission count rates for 186 keV energy line, in cps, *ε*_*186*_ is the detection efficiency at 186 keV, *ω*_*U*_ and *ω*_*Ra*_ are the emission probabilities for ^235^U and ^226^Ra photons at 185.7 and 186.2 keV, respectively, and *A*_*235U*_ is the ^235^U activity concentration derived from 143.8 keV line, in Bq kg^−1^. Such approach based on the mutual contribution of ^226^Ra and ^235^U is commonly practiced concerning determination of ^226^Ra isotope (Saïdou et al. 2008; El-Bahi et al. 2017).

**Table 2:** Activity concentrations of natural radionuclides related to bulk and fractionated phosphogypsum for PGA, PGB, and PGC samples; *n.f*. non-fractionated material

PG sample/isotope	Activity concentration, Bq kg^−1^ in fraction (fractions size in μm):					
	Bulk (n.f.)	F01 (< 63)	F02 (63–90)	F03 (90–125)	F04 (125–250)	F05 (> 250)
PGA (phosphogypsum derived from Syrian-Tunisian phosphorite blend, 1/1):						
^238^U (A_235U_·21.73)	266 ± 17	244 ± 16	239 ± 16	233 ± 16	298 ± 17	1320 ± 70
^235^U (143.8 keV)	12.3 ± 0.8	11.2 ± 0.8	11.0 ± 0.8	10.7 ± 0.7	13.7 ± 0.8	61 ± 3
^226^Ra (186.2 keV)	756 ± 23	1114 ± 25	657 ± 22	630 ± 22	660 ± 24	820 ± 80
^226^Ra (^214^Pb, ^214^Bi)	780 ± 80	1030 ± 130	670 ± 80	680 ± 70	660 ± 70	1160 ± 150
^210^Pb (46.5 keV)	796 ± 9	990 ± 10	787 ± 9	780 ± 9	848 ± 9	2270 ± 30
^228^Ra (^228^Ac)	23.2 ± 1.0	36 ± 6	19.9 ± 2.1	21 ± 5	25.7 ± 2.0	50 ± 10
^228^Th (^212^Pb, ^208^Tl)	3.7 ± 0.6	3.7 ± 1.4	5.6 ± 1.4	3.34 ± 0.26	7.0 ± 1.6	33 ± 9
PGB (phosphogypsum derived from Algerian-Tunisian phosphorite blend, 1/1):						
^238^U (A_235U_·21.73)	245 ± 16	156 ± 15	231 ± 16	330 ± 30	289 ± 17	1590 ± 80
^235^U (143.8 keV)	11.3 ± 0.7	7.2 ± 0.7	10.6 ± 0.7	15.4 ± 1.5	13.3 ± 0.8	73 ± 4
^226^Ra (186.2 keV)	592 ± 22	624 ± 21	502 ± 21	650 ± 40	707 ± 23	770 ± 90
^226^Ra (^214^Pb, ^214^Bi)	520 ± 50	530 ± 50	450 ± 50	560 ± 80	610 ± 70	940 ± 140
^210^Pb (46.5 keV)	491 ± 7	365 ± 7	439 ± 7	630 ± 14	547 ± 8	2370 ± 30
^228^Ra (^228^Ac)	43.1 ± 2.9	45 ± 5	39 ± 9	57 ± 7	53 ± 4	73 ± 8
^228^Th (^212^Pb, ^208^Tl)	10.9 ± 2.8	6.35 ± 0.24	8.8 ± 1.5	10.4 ± 2.7	15 ± 3	66 ± 13
PGC (phosphogypsum derived from Tunisian phosphorite):						
^238^U (A_235U_·21.73)	122 ± 14	142 ± 14	91 ± 13	420 ± 30	116 ± 13	270 ± 30
^235^U (143.8 keV)	5.6 ± 0.6	6.5 ± 0.7	4.2 ± 0.6	19.5 ± 1.4	5.3 ± 0.6	12.4 ± 1.5
^226^Ra (186.2 keV)	590 ± 20	560 ± 20	509 ± 18	390 ± 40	505 ± 18	670 ± 40
^226^Ra (^214^Pb, ^214^Bi)	540 ± 70	530 ± 50	460 ± 40	520 ± 60	480 ± 40	640 ± 70
^210^Pb (46.5 keV)	441 ± 7	416 ± 7	344 ± 7	609 ± 12	445 ± 7	841 ± 16
^228^Ra (^228^Ac)	47 ± 6	48 ± 4	39.1 ± 1.8	46 ± 7	42 ± 4	60 ± 8
^228^Th (^212^Pb, ^208^Tl)	8.2 ± 0.5	3.7 ± 0.5	4.3 ± 1.8	5 ± 3	5.8 ± 0.8	9 ± 3

The numerical data presented in Table 2 indicate that results of ^226^Ra activity concentrations obtained from this two-way approach generally converge in most cases of both non-fractionated and fractionated phosphogypsum samples; however, one can observe tendency toward obtaining slightly lower values for ^226^Ra activity concentration derived from ^214^Pb and ^214^Bi. This should be attributed to the possible ^222^Rn diffusion and escape from sealed samples containers. Thus, for the further analysis, ^226^Ra activity concentrations based on its 186.2-keV photons are discussed. The analysis of results included in Table 2 shows that activity concentration of ^226^Ra isotope in non-fractionated PGA, PGB, and PGC phosphogypsum samples vary from ca. 590 to 760 Bq kg^−1^. These are commonly observed values for phosphogypsum byproduct generated from phosphorites. In comparison, ^226^Ra concentration in phosphogypsum produced from apatite ores are found much lower (Msila et al. 2016; Grabas et al. 2018). The obtained results, regardless whether native or fractionated phosphogypsum is considered, clearly show that activity concentrations of ^226^Ra significantly exceed upper limit set for example by European Commission based on the 1.0 mSv a^−1^ dose criterion, assuming bulk application of phosphogypsum and its activity concentration index (European Commission 2000). The gamma spectrometry measurements revealed also moderate ^226^Ra concentration differentiation between particular grain size fractions among investigated phosphogypsum samples.

A useful parameter for a better recognition of the isotopes fractionation is an enrichment factor (*EF*), defined according to Eq. (6), as a ratio of the isotope activity in a particular fraction to that in non-fractionated material (Rutherford et al. 1996):

6 $$ EF=\frac{A_{Fi}}{A_{\mathrm{bulk}}} $$

where *A*_*Fi*_ and *A*_*bulk*_ are the activity of isotope in the fractionated material and in the bulk phosphogypsum, respectively, in Bq kg^−1^.

Taking into account results presented in Table 3, one can easily found that the overall deviations of ^226^Ra activity concentrations between particular fractions of phosphogypsum globally for PGA, PGB, and PGC samples related to the ^226^Ra concentration in non-fractionated material vary from − 34 to + 47% (enrichment factors from 0.66 up to 1.47). The highest deviations were observed in case of the Syrian-Tunisian phosphorite blend based phosphogypsum (PGA), and were within the range of 630 up to 1114 Bq kg^−1^ (enrichment factor between 0.83 up to 1.47) in case of the fractions F03 (90–125 μm) and F01 (below 63 μm), respectively. For the PGB phosphogypsum, ^226^Ra concentrations were between 502 and 770 Bq kg^−1^ in F02 (63–90 μm) and F05 (> 250 μm) fractions, respectively, what corresponds to the activity concentration between − 15 and + 30% in relation to the non-fractionated material (*EF* between 0.85 and 1.30). Even narrower range for ^226^Ra concentration was found for the purely Tunisian phosphorite based phosphogypsum (PGC), where enrichment factor varied between 0.66 and 1.14, corresponding to ^226^Ra concentration in the non-fractionated phosphogypsum between 390 and 670 Bq kg^−1^. It should be noted that among investigated set of PG samples, one can observe a slight tendency for enrichment of the coarse fractions with ^226^Ra. These results are in agreement with those previously reported in literature, where authors also reported enrichment of the fine fractions with ^226^Ra isotope (Rutherford et al. 1996; Kuzmanović et al. 2020).

**Table 3:** Average enrichment factors for ^238^U, ^226^Ra, ^210^Pb, and ^228^Th isotopes in different fractions of PGA, PGB, and PGC phosphogypsum samples

PG sample/isotope	Enrichment factor, *EF*, for isotope in fraction (in μm):				
	F01 (< 63)	F02 (63–90)	F03 (90–125)	F04 (125–250)	F05 (> 250)
PGA (phosphogypsum derived from Syrian-Tunisian phosphorite blend, 1/1):					
^238^U	0.92	0.90	0.88	1.12	4.97
^226^Ra	1.47	0.87	0.83	0.87	1.09
^210^Pb	1.24	0.99	0.98	1.07	2.85
^228^Th	0.98	1.50	0.89	1.88	8.89
PGB (phosphogypsum derived from Algerian-Tunisian phosphorite blend, 1/1):					
^238^U	0.64	0.94	1.37	1.18	6.50
^226^Ra	1.05	0.85	1.09	1.19	1.30
^210^Pb	0.74	0.89	1.28	1.11	4.83
^228^Th	0.58	0.81	0.95	1.37	6.03
PGC (phosphogypsum derived from Tunisian phosphorite):					
^238^U	1.17	0.75	3.48	0.95	2.21
^226^Ra	0.95	0.87	0.66	0.86	1.14
^210^Pb	0.94	0.78	1.38	1.01	1.91
^228^Th	0.45	0.53	0.64	0.71	1.16

The isotope with the second highest activity found in phosphogypsum is ^210^Pb. For this radionuclide activity, concentration varies between 441 and 796 Bq kg^−1^ for PGC and PGA samples, respectively. In the case of the fractionated material, similar behavior as for radium is observed and moderate ^210^Pb concentration fluctuations were found. The calculated enrichment factors varied between 0.74 and 1.38 for fractions F01 (< 63 μm, PGB) and F03 (90–125 μm, PGC), what corresponds to the overall ^210^Pb concentration range between 780 and 990 Bq kg^−1^ in the case of PGA, 365–630 Bq kg^−1^ for PGB, and 344–609 Bq kg^−1^ in the case of PGC fractionated samples (cf. Table 2). Significantly higher activity concentrations of ^210^Pb isotope are observed in case of the coarse fractions F05 (> 250 μm), where the measured activity concentrations are 2–4 times higher than in case of non-fractionated phosphogypsum. A similar behavior can be observed also for uranium isotopes, where activity concentrations of ^235^U and ^238^U isotopes in F05 fractions are 2.2–6.5 times higher than in non-fractionated materials (cf. data in Tables 2 and 3 for fractions F05 in PGB and PGC) as well as for ^228^Th isotope (activity concentration up to 8.9 times higher in case of PGA sample). One possible explanation of such behavior of lead, uranium, and thorium isotopes that is their much higher contribution in the coarse faction of phosphogypsum is their higher affinity to the organic material. As it was already mentioned, in case of investigated samples of phosphogypsum, the coarse fractions F05 (> 250 μm) of the materials contained clearly and easily identified organic contaminants, which can be responsible for selective accumulation of these isotopes during production process. It was already reported in the literature that these isotopes can be easily and effectively bound on the surface of organic particles (Mitchell et al. 2013). In the case of ^228^Ra isotope, observed behavior of this radionuclide in majority of cases follows ^226^Ra, but in practice, contribution of ^228^Ra to the total activity is negligible.

### Radioactive equilibrium and radionuclides fractionation in phosphogypsum

State of radioactive equilibrium is an excellent tool for studying radionuclides fractionation and their distribution in chemical processes, both in a laboratory and industrial scale. For sufficiently old raw materials, it can be easily assumed that naturally occurring decay chains are in secular equilibrium state. Among natural radioactive series of uranium (^238^U → → → ^206^Pb) and thorium chain (^232^Th → → → ^208^Pb), several isotopes may be selected for studying radionuclides fractionation phenomena. These can be such isotopic pairs as ^226^Ra/^238^U, ^222^Rn/^226^Ra, ^210^Pb/^226^Ra in case of uranium series as well as ^228^Ra/^232^Th, ^228^Th/^228^Ra or ^220^Rn/^224^Ra within thorium decay chain. Taking into account gamma spectrometry as an investigation tool, only few of these pairs are able to provide useful information about existing tendencies regarding particular elements and their behavior during chemical processing. Virtually, all chemical treatment applied on a material, in which radionuclides are in a radioactive equilibrium, would result in disruption of this state. Sometimes, as for example in case of ^222^Rn/^226^Ra, radioactive equilibrium can be already partially disrupted during simple mechanical operations (ore excavation and extraction, crushing, milling) due to the escape of the gaseous ^222^Rn isotope. Although, similar phenomena can occur in case of ^220^Rn/^224^Ra isotopic pair, in this circumstance, ^220^Rn escape is less likelihood due to the short half life of the thoron isotope.

Assuming typical partition coefficients for uranium and radium between phosphoric acid and phosphogypsum known from the literature (2.33 and 0.11 corresponding to 70% retention of ^238^U in phosphoric acid and 90% of ^226^Ra in PG, lead behavior is very similar to Ra) and taking into account measured ^238^U, ^226^Ra, and ^210^Pb activity concentrations in the phosphogypsum phase (cf. Table 2), one can estimate radionuclides activities in phosphorite ore used and in formed phosphoric acid (Mazzilli et al. 2000; Santos et al. 2006; Bolívar et al. 2009).

In the case of phosphogypsum residue, analysis of the radioactive equilibrium is effective and effortless for ^238^U series isotopes, due to their high concentrations in phases of phosphorite ore (PO), formed phosphoric acid (PA), and phosphogypsum (PG). Simplification of the ^238^U decay chain allows to write Eq. (7) for the main isotopes considered as the radioactive equilibrium state indicators: ^238^U, ^226^Ra and ^210^Pb.

7 $$ {}^{238}U\to \dots \to {}^{226} Ra\to \dots \to {}^{210} Pb $$

**Fig. 2:**
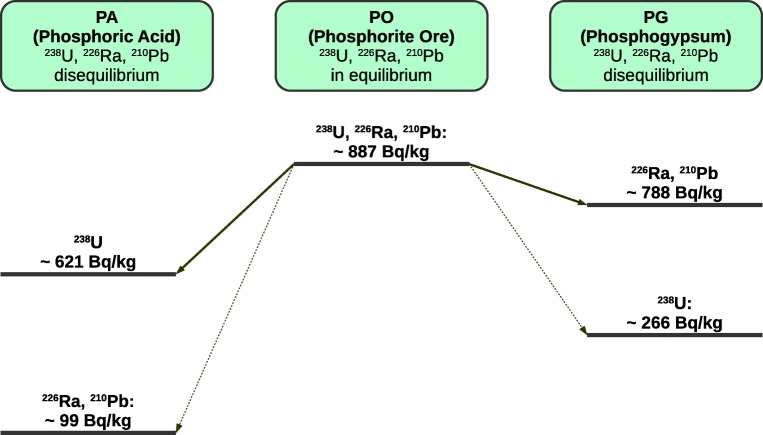
Partitioning of main ^238^U series radionuclides between phosphogypsum (PG) and phosphoric acid (PA) phase for PGA phosphogypsum sample; calculations performed assuming 70% retention of uranium in PA and 90% accumulation of ^226^Ra in phosphogypsum (thick continuous line – main streams, thin dashed line – minor material streams)

Figure 2 presents results of such analysis for the ^238^U, ^226^Ra, and ^210^Pb activity concentrations in the PGA phosphogypsum sample and related concentrations in PA and PO. All concentrations have been related to phosphogypsum phase. In secular equilibrium conditions, activity concentration of each particular radionuclide in a decay chain is the same, what can be expressed by Eq. (8):

8 $$ {A}_{238U}={A}_{226 Ra}={A}_{210 Pb} $$

where *A*_*238U*_, *A*_*226Ra*_ and *A*_*210Pb*_ are activity concentrations of the ^238^U, ^226^Ra, and ^210^Pb radionuclide, in Bq kg^−1^. The data presented in Fig. 2 clearly show the existing disequilibrium between ^238^U and ^226^Ra isotopes in PG and PA. It is easy to deduce that distribution ratio for each radionuclide must be dependent on the chemical form of the isotope in the reaction mixture and its affinity to the phases being formed during chemical processing. Taking into account secular equilibrium conditions at the beginning of the process resulting in a constant activity ratio for all isotopic pairs in a decay series equal 1, after processing, particular activity ratios can depart from the unity: below one when fraction is depleted and over one when fraction is enriched in a daughter isotope.

### Isotopes activity ratios in fractionated PG

The isotopic activity ratio can be a useful indicator for the radionuclides partitioning between different fractions of phosphogypsum. The possible isotopic pairs for possible determination by gamma-ray spectrometry and with relatively long half-lives are ^226^Ra and ^238^U as well as ^210^Pb and ^226^Ra in uranium chain, and ^228^Th and ^228^Ra radionuclides within the thorium series.

**Fig. 3:**
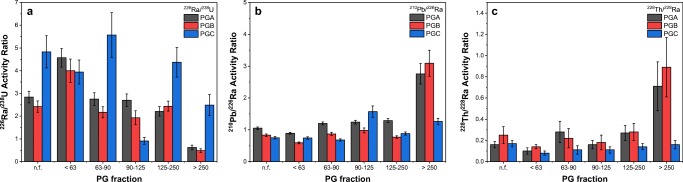
Activity ratios of ^226^Ra and ^238^U (**a**), ^210^Pb and ^226^Ra (**b**), and ^228^Th and ^228^Ra (**c**) in different fractions of phosphogypsum in PGA, PGB, and PGC samples

Taking into account activity concentrations presented already in Table 2, one can easily calculate activity ratios for ^226^Ra/^238^U, ^210^Pb/^226^Ra, and ^228^Th/^228^Ra. The results of these calculations are presented in Table 4. Further, Fig. 3 allows for convenient comparison of the obtained values. As can be found, there are no clear tendencies for activity ratios of particular isotopic pairs ^226^Ra/^238^U, ^210^Pb/^226^Ra, and ^228^Th/^228^Ra among three analyzed phosphogypsum samples. Figure 3a presents results of ^226^Ra/^238^U activity ratio for native and fractionated phosphogypsum PGA, PGB, and PGC. It is easy to find that already for non-fractionated samples, ^226^Ra and ^238^U activity ratios differ significantly from 2.42 (PGB) up to 4.8 (PGC). It is obvious that presented figures indicate clearly for enrichment of phosphogypsum phase with ^226^Ra isotope, but the observed enrichment can be variable. Here, reasonably assuming radioactive equilibrium between ^226^Ra and ^238^U isotopes in phosphorite ore and initial activity ratio of ^226^Ra/^238^U equals to 1, the phosphogypsum phase accepts from 71 up to even 83% of total ^226^Ra activity (what can be easily deduced from for ^226^Ra/^238^U activity ratio of respectively 2.42 and 4.8). These figures were already confirmed in literature (Al-Hwaiti et al. 2010b).

**Table 4:** Activity ratios of radionuclides from uranium and thorium series in different phosphogypsum fractions

Sample/fraction (fraction size in μm)	Activity ratio		
	^226^Ra/^238^U	^210^Pb/^226^Ra	^228^Th/^228^Ra
PGA (phosphogypsum derived from Syrian-Tunisian phosphorite blend, 1/1):			
bulk (n.f.)	2.84 ± 0.26	1.05 ± 0.04	0.16 ± 0.03
F01 (< 63)	4.6 ± 0.4	0.89 ± 0.03	0.10 ± 0.03
F02 (63–90)	2.75 ± 0.28	1.20 ± 0.05	0.28 ± 0.10
F03 (90–125)	2.70 ± 0.28	1.24 ± 0.06	0.16 ± 0.04
F04 (125–250)	2.21 ± 0.21	1.29 ± 0.06	0.27 ± 0.07
F05 (> 250)	0.62 ± 0.10	2.8 ± 0.3	0.7 ± 0.3
PGB (phosphogypsum derived from Algerian-Tunisian phosphorite blend, 1/1):			
bulk (n.f.)	2.42 ± 0.25	0.83 ± 0.04	0.25 ± 0.08
F01 (< 63)	4.0 ± 0.5	0.59 ± 0.03	0.140 ± 0.019
F02 (63–90)	2.17 ± 0.24	0.87 ± 0.05	0.22 ± 0.09
F03 (90–125)	1.9 ± 0.3	0.98 ± 0.08	0.18 ± 0.07
F04 (125–250)	2.44 ± 0.22	0.77 ± 0.04	0.28 ± 0.08
F05 (> 250)	0.48 ± 0.08	3.1 ± 0.4	0.89 ± 0.28
PGC (phosphogypsum derived from Tunisian phosphorite):			
bulk (n.f.)	4.8 ± 0.7	0.75 ± 0.04	0.17 ± 0.03
F01 (< 63)	3.9 ± 0.5	0.74 ± 0.04	0.077 ± 0.017
F02 (63–90)	5.6 ± 1.0	0.68 ± 0.04	0.11 ± 0.04
F03 (90–125)	0.91 ± 0.15	1.57 ± 0.18	0.11 ± 0.03
F04 (125–250)	4.4 ± 0.7	0.88 ± 0.05	0.14 ± 0.03
F05 (> 250)	2.5 ± 0.5	1.26 ± 0.10	0.16 ± 0.04

Much wider range of ^226^Ra/^238^U activity ratio is observed in fractionated phosphogypsum. Its values found for investigated samples are between 0.48 (PGB, fraction F05, > 250 μm) up to 5.6 (PGC, fraction F02, 63–90 μm). For the fractionated phosphogypsum samples, it was found that there is a slight trend for decreasing of ^226^Ra enrichment factor with increasing grain size of the materials. This tendency is confirmed by the data presented in Fig. 3a, although absolute values of the ^226^Ra/^238^U activity ratio vary significantly.

For ^210^Pb/^226^Ra activity ratio in non-fractionated material, one could observe values close to or slightly below 1, what suggests similar behavior of lead and radium isotopes during chemical processing of the phosphorite ore. The presented values in Table 4 and Fig. 3b differ slightly but are within the range 0.75–1.05 for PGC and PGA samples respectively. These values of ^210^Pb/^226^Ra activity ratio suggest, although similar chemical properties and crystal ionic radii for both ions (133 pm for Pb^2+^ and 162 pm for Ra^2+^), for slightly higher preference of radium retention in phosphogypsum in comparison with lead. Further analysis of ^210^Pb/^226^Ra activity ratio for fractionated phosphogypsum reveals that for the larger grain size, this preferential trend for radium accumulation in phosphogypsum decreases toward increasing lead contribution in PG. This is clearly confirmed by direct comparison of ^210^Pb/^226^Ra activity ratios among analyzed phosphogypsum samples for PGA, PGB, and PGC (cf. Table 4 and Fig. 3b). The highest lead enrichment factors (but except PGC sample) are observed for fractions F05 (> 250 μm), and this effect can be explained by the presence of traces of organic matter. In such case, significant part of equilibrium polonium activity (^218^Po and ^214^Po) is retained within the organic matter containing phase and due to a relatively fast conversion by decay to ^210^Pb increase final lead activity in the coarse fraction. Moreover, one has to take into account lower ^222^Rn exhalation rate from the coarse phosphogypsum fractions. Larger grain sizes led to a lower surface area and decreased ^226^Ra fraction available for exhalation, what in effect results in increased contribution of ^210^Pb in coarse phosphogypsum fractions.

Last indicator, based on ^228^Th/^228^Ra activity ratio provides information on thorium and radium distribution among different fractions of phosphogypsum. It was found, that among analyzed fractions, this parameter is relatively stable within the PG grain sizes up to 250 μm. The results included in Table 4 and Fig. 3b indicate that ^228^Th/^228^Ra activity ratios are within the ranges of 0.10–0.28 for PGA, 0.14–0.28 for PGB, and 0.08–0.17 for PGC. Only in case of the coarse fraction (F05, > 250 μm), ^228^Th/^228^Ra activity ratio increases significantly up to 0.7 for PGA and even 0.89 for PGB samples, while for PGC phosphogypsum remains at a constant level of 0.16, similar to the ^228^Th/^228^Ra activity ratio in the finer fractions. These results obtained for ^228^Th and ^228^Ra isotopic pair confirm preferential accumulation of radium in phosphogypsum; however, again one can conclude that in the coarse fraction, this tendency is strongly interfered probably by the presence of the organic matter contaminants, which are able to adsorb preferentially thorium.

### Factors affecting radionuclides fractionation during PG production

**Fig. 4:**
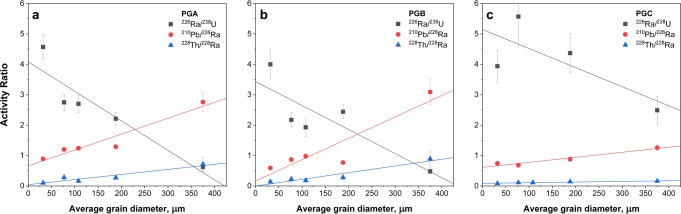
Correlation of the average grain size with activity ratios of ^226^Ra/^238^U, ^210^Pb/^226^Ra, and ^228^Th/^228^Ra isotopic pairs in PGA (**a**), PGB (**b**), and PGC (**c**) phosphogypsum samples

The data presented in Fig. 4a–c shows dependencies between the observed activity ratios of ^226^Ra/^238^U, ^210^Pb/^226^Ra and ^228^Th/^228^Ra isotopic pairs and average grain diameter of phosphogypsum. As can be seen, significant correlations exist for all three isotopic pairs analyzed, and coefficients of determination (*R*^*2*^) based on PGA and PGB phosphogypsum samples are found to be 0.86 and 0.72 for ^226^Ra/^238^U (Fig. 4a, b), for ^210^Pb/^226^Ra 0.92, 0.84, and 0.94 respectively for PGA, PGB, and PGC (after discarding of one bad data point), and finally for ^228^Th/^228^Ra within the range 0.88–0.92 for PGA, PGB and PGC samples. Only in case of ^226^Ra/^238^U ratio for PGC sample, coefficient of determination departs significantly from unity (*R*^*2*^ = 0.09), but this is due to the anomalous data point identified for 90–125 μm grain size (cf. Table 4 and Fig. 4c). After discarding the outlier value, the coefficient of determination takes value of *R*^*2*^ = 0.57, also indicating for a strong correlation between ^226^Ra/^238^U isotopic ratio vs. average particles grain diameter.

In general, one should note that all dependencies presented in Fig. 4a–c exhibit similar behavior. It is obvious that there exists very clear propensity of radium to locate preferentially in the smaller grain size fractions. Oppositely, lead and thorium exhibit higher tendency to be incorporated in the more coarse fractions. Because natural radionuclides in phosphorite ores, phosphoric acid, and phosphogypsum exist in very low concentrations, they must be considered as trace elements and their behavior is governed by rules related to microcomponents. In such case, radium will be concentrated in particular phases together with the carriers, present in the reaction mixture in higher concentrations. Thus, radium retention in a gypsum must be a result of its propensity to coprecipitate with calcium sulfate and the solubility product of CaSO_4_, and is accompanied by a presence of other sulfate forming insoluble ions like Ba^2+^ and Sr^2+^ (Rutherford et al. 1996).

To get a more detailed insight into above findings, one should consider also the simplest mode of ions incorporation into a crystal lattice, which is an interstitial incorporation. In this mechanism, a foreign ion is accommodated at the interstitial position between the host lattice ions. However, the very common mechanism for the trace components is coprecipitation, which occurs if a tracer and oppositely charged ion of precipitate are able to form isomorphous crystals with the precipitate. Then, the tracer ion is incorporated into precipitate crystal lattice, where substitutes the main cation, and process is especially favorable when the tracer and the cation forming precipitate are close in sizes. But in the case of Ca^2+^ and Ra^2+^, this condition is not fulfilled. This is because crystal ionic radii of calcium and radium ions differ significantly and are 114 and 162 pm respectively. Additionally, calcium and radium sulfates do not form isomorphous crystals: calcium sulfate crystallizes as CaSO_4_·2H_2_O in a monoclinic system whereas radium sulfate as RaSO_4_ in an orthorhombic system. The same discrepancy is found for lead sulfate (PbSO_4_) and thorium sulfate (Th(SO_4_)_2_·9H_2_O), but here, the basic difference between these compounds is that thorium sulfate can be considered as a soluble salt. Due to these reasons, radium is not efficiently incorporated into gypsum lattice in comparison with processes in which isomorphous crystals are formed, as BaSO_4_, PbSO_4_ or SrSO_4_, what was already confirmed in literature (Matyskin et al. 2017). It was reported that distribution coefficient between gypsum and aqueous phase for radium is generally low and according to different sources between 0.03 and 0.3 (Lestini et al. 2013, 2019). From the other hand, production of phosphoric acid by means of the wet method based on the phosphorite ores is burdened by the presence of other trace elements and contaminants, which actually exist in much higher concentrations in the reaction slurry than radioactive contaminants. It was already reported that incorporation of radium into gypsum phase is much more efficient when contaminants, as e.g., Sr^2+^, are present (Lestini et al. 2019). The presence of the contaminants favors formation of isomorphous with RaSO_4_ phases (e.g., SrSO_4_, BaSO_4_, PbSO_4_) next to the CaSO_4_·2H_2_O formation. One should expect that these foreign phases are formed independently to the main calcium sulfate product and remain in the form of smaller particles in comparison with the main precipitate. As contribution of the main product increases in time, radium bearing phases become incorporated into major phase forming conglomerates. The possible explanation for the observed effects regarding preferential incorporation of radionuclides into different grain size fraction must involve all discussed above factors. The major mechanisms of foreign ions incorporation into phosphogypsum are interstitial incorporation, coprecipitation, or isomorphous substitution followed by particles conglomeration. It is obvious that when moving from a fine to the coarse fraction of the material, simultaneously specific surface area of the material decreases, and from the presented data, it is evident that radionuclides ions incorporation must be straightly related to the surface area of the phosphogypsum crystals formed. This gives the presumption that for ^226^Ra incorporation into gypsum phase, mechanism of incorporation must be in a sequence of radium bearing sulfate phases formation followed by surface adsorption of these phases on a calcium sulfate crystals. Contrary to that, for lead and thorium ions, rather incorporation into crystal lattice should be expected as more likelihood mechanism. A phosphogypsum formation process is a dynamic phenomenon, in which the foreign ions incorporation into calcium sulfate phase occurs during crystals growth. Since in the industrial scale the growth does never take place at equilibrium conditions, radionuclides incorporation into phosphogypsum is a complex mechanism affected by many factors.

## Conclusions

The main goal of this work was to find out if there is any rational possibility to separate phosphogypsum into fractions with different radium content. Presented results clearly show that there is no significant differentiation between ^226^Ra distribution among particular grain size fractions of this material; however, obvious tendency for preferential retention of radionuclides in particular grain size fractions is observed. The secondary object of this work was an analysis of the existing disequilibrium between main radioactive contaminants in phosphogypsum and their progenies used for quantification of radionuclides in PG. The results presented in this paper show that isotopic ratios of ^226^Ra/^238^U, ^210^Pb/^226^Ra, and ^228^Th/^228^Ra can be a useful tool for studying radionuclides fractionation mechanisms, and gamma spectrometry method used in this study requires only minimal laboratory work. Moreover, presented results clearly prove that in chemically modified materials, the radioactive equilibrium between particular parents and daughters isotopes is disrupted and one has to be very careful when using gamma spectrometry technique for determination of natural radionuclides in such materials. It is especially important regarding ^232^Th isotope, which cannot be directly determined using gamma spectrometry and is usually measured assuming radioactive equilibrium. The results show that in the case of thorium isotopes, the radioactive equilibrium conditions assumed between ^232^Th, ^228^Ra, and ^228^Ac very often does not occur. All three discussed in this work examples of phosphogypsum residues are far away from equilibrium and direct determination of ^232^Th by gamma spectrometry is in practice impossible. This is surely valid also for other industrial byproducts, waste, and residues from chemical industry, where any isotopes separation may occur.
